# 

**Published:** 1897-04

**Authors:** 


					﻿ATLANTA
MEDICAL AND SURGICAL
_______JOURNAL.
NEW sebmb. Atlanta, Ga., April, 1897. No. 2.
				

